# Anatomical variation of the sacroiliac joints: an MRI study with synthetic CT images

**DOI:** 10.1186/s13244-023-01373-1

**Published:** 2023-02-08

**Authors:** Elke Vereecke, Lieve Morbée, Frederiek Laloo, Min Chen, Jacob L. Jaremko, Nele Herregods, Lennart Jans

**Affiliations:** 1 Department of Radiology, Ghent University Hospital, Corneel Heymanslaan 10, 9000 Ghent, Belgium; 2 Department of Radiology, Peking University Shenzhen Hospital, Shenzhen, 518036 China; 3 Department of Radiology and Diagnostic Imaging, University of Alberta Hospital, Edmonton, AB T6G 2B7 Canada

**Keywords:** Sacroiliac joint, Anatomic variation, Magnetic resonance imaging, Artificial intelligence, Synthetic computed tomography

## Abstract

**Background:**

Synthetic computed tomography (sCT) images are magnetic resonance imaging (MRI)-based images, generated using artificial intelligence. This study aimed to determine the prevalence of anatomical variants of sacroiliac joints (SIJ) on sCT images and the correlation with age, sex and body weight.

**Methods:**

MRI of the SIJ including sCT images of 215 patients clinically suspected for sacroiliitis were retrospectively analyzed. The presence of anatomical variants of the SIJ was assessed. Age, sex and body mass index at the time of the MRI were recorded.

**Results:**

SIJ variants were found in 82.8% (356/430) of the evaluated joints. The most frequent variants were iliosacral complex (27.7%), bipartite iliac bony plate (27.2%) and crescent iliac bony plate (27%). One new variant was identified, consisting of an accessory facet of the SIJ on the superior side. Overall, SIJ variants were slightly more frequent in women (85.8% vs. 77.8%), but iliosacral complex was significantly more frequent in men. Isolated synostosis was more prevalent with advancing age, in contrast to semicircular defect and unfused ossification center. The occurrence of iliosacral complex was associated with higher BMI, while crescent iliac bony plate occurred more in patients with lower BMI.

**Conclusion:**

Over 80% of patients in this study, who were all suspected of sacroiliitis, had at least one SIJ variant. These variants may actually represent subtypes of the normal SIJ. sCT enables detection of very small or subtle findings including SIJ variants.

## Key points

1. Sacroiliac joints (SIJ) variants are very commonly observed in patients suspected of sacroiliitis.
2. Synthetic CT allows detection of small or subtle findings including SIJ variants.
3. Women have a higher prevalence of SIJ variants.
4. Multiple variants can coexist within one SIJ.
5. High prevalence of variants indicates existence of subtypes of the ‘normal’ SIJ.

## Introduction

The sacroiliac joint (SIJ) is a complex anatomical structure. Multiple anatomical variants of the SIJ have been described [1–11]. Different factors have been suggested regarding their etiology, including congenital or hereditary factors, as well as the influence of (altered) mechanical stress [2, 4, 5, 11].

SIJ variants are variations in the morphology of the osseous structures of the sacrum and the ilium. As computed tomography (CT) excels in depicting bone, most studies describing these variants have been conducted on CT [1, 2, 4–9, 11]. However, MRI is the imaging modality of choice to examine the SIJ for sacroiliitis, as it allows detection of bone marrow edema, a key characteristic of active inflammatory disease on MRI [12]. Furthermore, edema and/or structural changes have been described in some SIJ variants [1, 3–6, 9, 10], as well as associations with symptomatic disease, indicating their potential clinical relevance [1, 4, 8]. Moreover, El Rafei et al. pointed out that these edematous or structural alterations could be mechanical in nature, and they should not be mistaken for inflammatory sacroiliitis [3]. Knowledge of these variants is therefore essential for a correct interpretation of MRI studies of SIJ.

Synthetic CT (sCT) uses artificial intelligence to generate CT-like images derived from MRI sequences [13]. This allows for excellent visualization of the bony structures without use of potential harmful ionizing radiation, while also obtaining conventional MR images in the same examination. Moreover, these sCT images allow for postprocessing with multiplanar reconstruction, which is practical and useful when examining the complex anatomical structure of the SIJ. This technique has been clinically validated in the SIJ, hips, lumbar spine and cervical spine [13–19]. In a recent study by Jans et al. [14], sCT outperformed T1-weighted MRI images for detection of erosions, sclerosis, and ankylosis of the SIJ in patients with axial spondyloarthritis (axSpA), with reliability comparable to that of CT. In addition to the SIJ, the reliability of sCT in comparison with conventional CT has been demonstrated for the pelvis including hips [15, 17, 18], lumbar spine [16], and cervical spine [19]. We aimed to evaluate the prevalence of SIJ variants on sCT images in a group of patients clinically suspected of axial axSpA, and to analyze the relationship between SIJ variants and age, sex and body weight. Finally, we sought to describe the coexistence of multiple variants within one SIJ.

## Methods

### Study patients

Patients who had undergone MRI of the sacroiliac joints including sCT in our hospital between 05/02/2019 and 05/02/2022 were retrospectively included. All patients were referred to a tertiary hospital with clinical suspicion of sacroiliitis. Age, sex and body mass index (BMI) at the time of the MRI were recorded. BMI was subdivided into groups according to the World Health Organization International Classification [20]: below 18.5 was defined as underweight, 18.5 to 24.9 as healthy, 25.0 to 29.9 as overweight and 30.0 and above as obese. Exclusion criteria consisted of age less than 18 at the time of the MRI.

### Image acquisition

All MR studies were performed on a 3.0 T MR unit (Prisma, Siemens Healthineers). Routine MRI protocol of the SIJ included semi-coronal (along the long axis of the sacrum) T1-weighted turbo spin echo imaging (slice thickness (ST) 3 mm; repetition time/echo time (TR/TE): 559/9.9 ms), semi-coronal short tau inversion recovery imaging (STIR) (ST 3 mm; TR/TE/inversion time 4600/38/220 ms) and axial short tau inversion recovery imaging of the pelvis (ST 4 mm; TR/TE/inversion time: 8190/57/220 ms). For sCT reconstruction with the commercially available software BoneMRI Pelvic region (version 1.4, MRIguidance BV), an axial 3D T1-weighted radio-frequency-spoiled multiple gradient echo sequence was scanned (2 echoes: TR/TE1/TE2: 7/2/3.53 ms, field of view: 400 × 400 mm, reconstructed voxel size: 0.52 × 0.52 × 0.8 mm, acquisition time: 4 min 43 s). Reconstruction of sCT images runs automatically: the sCT images are available as a 3D volume in axial plane in the hospital picture archiving and communication system (PACS) after a processing time of 30 min. Relevant scanning parameters are summarized in Table 1.

**Table 1:** Technical parameters of the MRI sequences

	Semi-coronal TSE T1	Semi-coronal STIR	Axial STIR	Axial 3D T1-weighted radio-frequency-spoiled multiple GRE
Location	Sacrum and SIJ	Sacrum and SIJ	Pelvis	Pelvis
Slice thickness (mm)	3	3	4	N/A
Repetition time (ms)	559	4600	8190	7
Echo time 1 (ms)	9.9	38	57	2
Inversion time (ms)	N/A	220	220	N/A
Echo time 2 (ms)	N/A	N/A	N/A	3.53
Field of view (mm)	220 × 220	220 × 220	380 × 380	400 × 400
Voxel size (mm)	0.6 × 0.6 × 3.0	0.4 × 0.4 × 3.0	1.0 × 1.0 × 4.0	0.52 × 0.52 × 0.8
Acquisition time	3 m 24 s	3 m 10 s	3 m 02 s	4 m 43 s

MRI = Magnetic resonance imaging; TSE = turbo-spin echo; STIR = short tau inversion recovery; GRE = gradient echo; SIJ = sacroiliac joints; ms = millisecond; mm = millimeter; N/A = not applicable; m = minutes; s = seconds

### Image analysis

Two radiologists with 9 and 8 years of experience in musculoskeletal radiology scored the images independently, after scoring 10 cases in consensus as a calibration exercise. A consensus reading between both readers was done in case of discrepancies, to generate final scores.

For each SIJ, the presence of anatomical variants of the SIJ was assessed, for the cartilaginous as well as the ligamentary part of the SIJ.

All anatomical characteristics were determined based on the sCT images, and dynamic multiplanar reconstruction (available as a tool in PACS) was allowed. No paired CT data were used as this imaging technique has been clinically validated by several previous studies [13–19].

The quality of the sCT images was very good to excellent for all cases, and there were no images with artifacts that would have impaired image assessment.

### Definitions

**Fig. 1:**
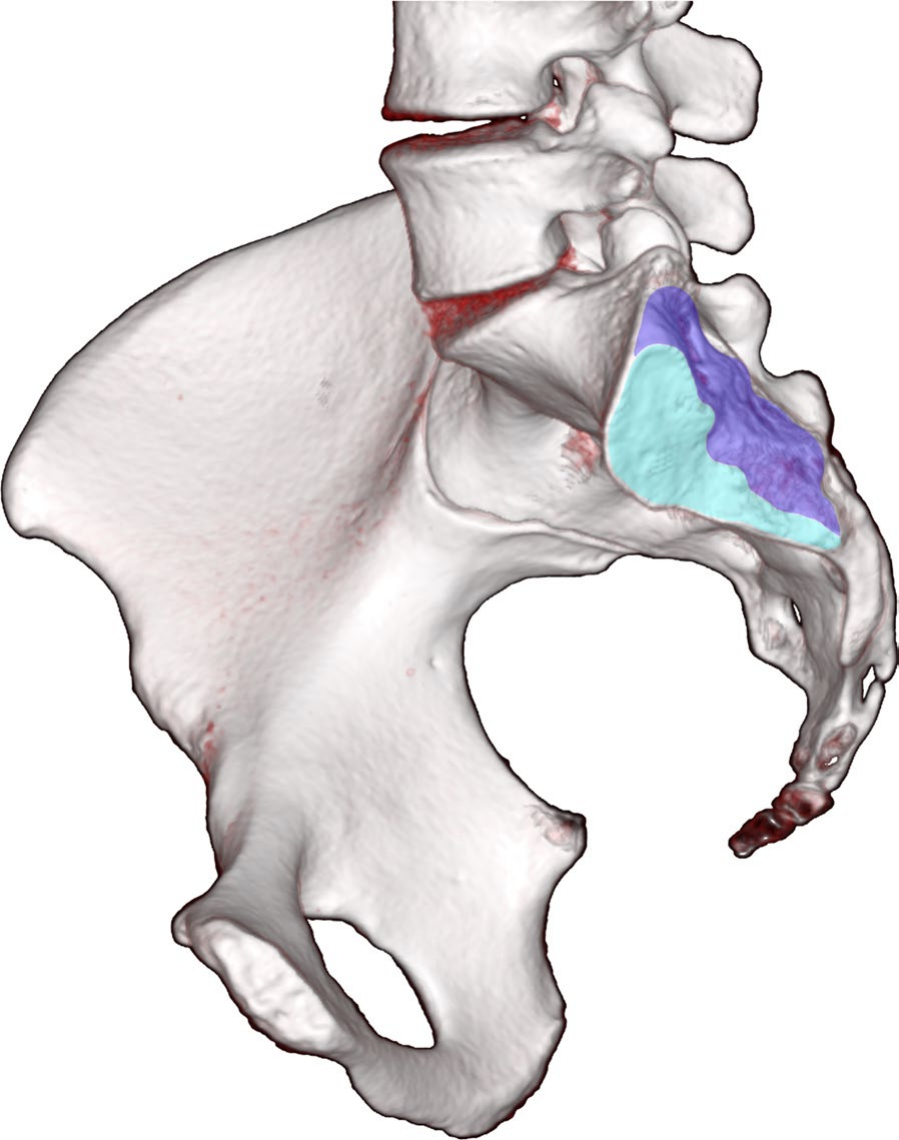
Illustration of sacroiliac joint. In this illustration, the sacroiliac joint is divided into an anterior cartilaginous part (light blue) and a more posteriorly located ligamentous part (dark blue)

Anatomical variants of the SIJ were defined using the classification by Prassopoulos et al. [4] supplemented by other forms recognized in an MRI-based study by El Rafei et al. [3] The SIJ was divided into an anterior-inferior cartilaginous part, and a posterior-superior ligamentary part (Fig. 1) [21].

The different variants in the cartilaginous part are:

- Unfused ossification center, with a separate often triangular osseous structure anterosuperiorly to the SIJ,
- Focal dysmorphic sacrum, formed by a prominent ridge of the posterior part of the sacral surface of the joint, protruding into the iliac bone,
- Isolated synostosis with focal bony bridging,

The variants of the ligamentary part consisted of:

- Bipartite iliac bony plate, with a division in the posterior part of the iliac part of the SIJ,
- Accessory joint posterior to the cartilaginous part of the joint,
- Iliosacral complex with a prominent convex notch and a corresponding sacral groove,
- Semicircular defect where there is a round defect in the sacrum and sometimes also in the overlying ilium,
- Crescent iliac bony plate where the normal overall convex ilium is concave, with or without bulging of the sacral surface.

**Fig. 2:**
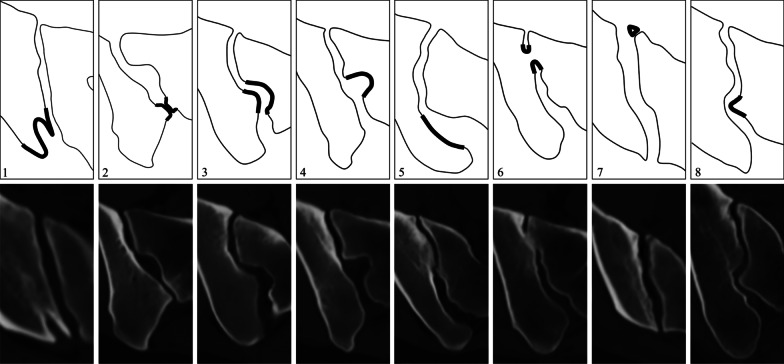
Schematic drawing and corresponding synthetic CT images of sacroiliac joint (SIJ) variations, all shown in semi-axial plane: (1) bipartite iliac bony plate, (2) accessory SIJ, (3) iliosacral complex, (4) semicircular defect, (5) crescent ilium, (6) isolated synostosis, (7) unfused ossification center, and (8) dysmorphic sacrum

Schematic drawings and sCT imaging examples of these SIJ variants are provided in Fig. 2.

### Statistical analysis

For each variant, differences in proportion of that variant between males and females were assessed by means of generalized estimating equations (GEE), taking the clustered nature of joints within each patient into account. Association between occurrence of SIJ variants and age and BMI was investigated in the same way, and was reported by odds ratio (OR) and 95% confidence interval (CI) based on GEE analysis. Prevalences of joint variants, including uni- or bilateral occurrence and combinations of variants, were analyzed descriptively. The inter-observer agreement for detection of SIJ variants on sCT images was assessed using kappa (K) statistics [22]. Analyses were performed using statistical software SPSS (version 28). *p* value < 0.05 was considered statistically significant.

## Results

### Patient characteristics

The population consisted of 215 participants (81 men and 134 women). The mean age was 37 ± 10.6 years. Mean BMI was 25.1 ± 4.6. Six patients had underweight, 111 patients had a healthy weight, 64 were overweight and 34 were obese.

### Occurrence of variants and association with sex, age and BMI

**Fig. 3:**
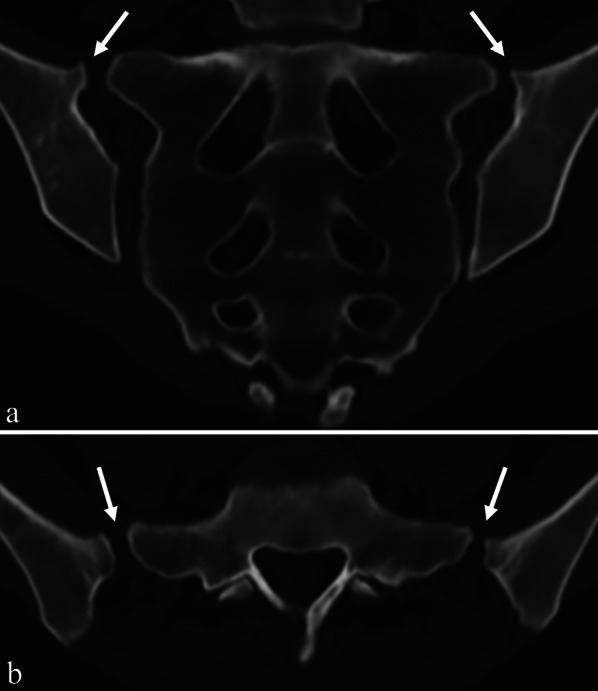
New sacroiliac joint (SIJ) variant with extra joint facet superiorly. Semi-coronal (**a**) and semi-axial (**b**) synthetic CT images depict a newly found SIJ variant in a 27-year-old women, consisting of an accessory joint cranial of the cartilaginous part of the SIJ, between bony projections from the ilium (arrows) and the posterosuperior edge of S1 vertebra

A total of 430 SIJ were evaluated on sCT images. At least one variant was detected in 356 (82.8%) of all evaluated joints. The prevalence of SIJ variants according to sex, age and BMI is shown in Table 2. Iliosacral complex was the most common variant (27.7%), closely followed by bipartite iliac bony plate (27.2%) and crescent iliac bony plate (27%). The least frequent variants were isolated synostosis and unfused ossification center, found in 2.1% and 0.7%, respectively. We found a new variant, present bilaterally in one patient, consisting of an extra joint facet on the superior side of the cartilaginous part of the SIJ (Fig. 3). This variant resembles the typical accessory joint, but is found in a different location; while the accessory SIJ is located in the ligamentous part of the SIJ, posteriorly to the cartilaginous part of the SIJ, this new variant is found on the cranial side of the SIJ, cranially to the cartilaginous part of the SIJ.

**Table 2:** Frequencies of SIJ variants according to sex, age and BMI

	Total study group	Sex		Age (years)							BMI (kg/m^2^)*			
		Male	Female	18–20	21–30	31–40	41–50	51–60	61–70	71–80	< 18.5	18.5–24.9	25–29.9	≥ 30
Total number of joints	430	162	268	22	104	140	112	44	6	2	12	222	128	68
No variant	74 (17.2%)	36 (22.2%)	38 (14.2%)	3 (13.6%)	20 (19.2%)	26 (18.6%)	19 (17%)	4 (9.1%)	2 (33.3%)	0	2 (16.7%)	42 (18.9%)	21 (16.4%)	9 (13.2%)
Bipartite iliac bony plate	117 (27.2%)	13 (8%)	104 (38.8%)	6 (27.3%)	26 (25%)	47 (33.6%)	25 (22.3%)	11 (25%)	2 (33.3%)	0	5 (41.7%)	63 (28.4%)	33 (25.8%)	16 (23.5%)
Accessory joint	56 (13%)	14 (8.6%)	42 (15.7%)	1 (4.5%)	11 (10.6%)	26 (18.6%)	10 (8.9%)	4 (9.1%)	2 (33.3%)	2 (100%)	2 (16.7%)	22 (9.9%)	24 (18.8%)	8 (11.8%)
Iliosacral complex	119 (27.7%)	64 (39.5%)	55 (20.5%)	2 (9.1%)	35 (33.7%)	28 (20%)	32 (28.6%)	22 (50%)	0	0	4 (33.3%)	47 (21.2%)	40 (31.3%)	28 (41.2%)
Semicircular defect	34 (7.9%)	10 (6.2%)	24 (9%)	4 (18.2%)	7 (6.7%)	18 (12.9%)	5 (4.5%)	0	0	0	0	19 (8.6%)	7 (5.5%)	8 (11.8%)
Crescent iliac bony plate	116 (27%)	39 (24.1%)	77 (28.7%)	10 (45.5%)	24 (23.1%)	43 (30.7%)	27 (24.1%)	10 (22.7%)	2 (33.3%)	0	6 (50%)	69 (31.1%)	31 (24.2%)	10 (14.7%)
Isolated synostosis	9 (2.1%)	6 (3.7%)	3 (1.1%)	0	0	1 (0.7%)	5 (4.5%)	1 (2.3%)	0	2 (100%)	0	6 (2.7%)	3 (2.3%)	0
Unfused ossification center	3 (0.7%)	2 (1.2%)	1 (0.4%)	0	2 (1.9%)	1 (0.7%)	0	0	0	0	0	1 (0.5%)	2 (1.6%)	0
Dysmorphic posterior part	65 (15.1%)	9 (5.6%)	56 (20.9%)	2 (9.1%)	10 (9.6%)	21 (15%)	29 (25.9%)	3 (6.8%)	0	0	4 (33.3%)	30 (13.5%)	21 (16.4%)	10 (14.7%)
Extra SIJ facet superiorly	2 (0.5%)	0	2 (0.7%)	0	2 (1.9%)	0	0	0	0	0	0	0	0	2 (2.9%)

Values are N (number of observations), with column percentages in parentheses. Note that in some columns the percentages do not add up to 100% because some SIJ demonstrated multiple variants

*BMI was subdivided into groups according to the WHO International Classification [13]: underweight (below 18.5), healthy (18.5 to 24.9), overweight (25.0 to 29.9) and obese (30.0 and above)

SIJ = sacroiliac joint, BMI = body mass index

Variants were more often found in women (85.8%) than men (77.8%), except for iliosacral complex, and the more rare variants isolated synostosis and unfused ossification center. Bipartite iliac bony plate and dysmorphic SIJ were found significantly more frequently in women (*p* < 0.001 and 0.003 respectively), whereas iliosacral complex occurred more frequently in men (*p* = 0.003) (Table 3).

**Table 3:** Association of SIJ variants with sex, age and BMI

	Sex	Age	BMI
	OR [95% CI]; *p* value	OR [95% CI]; *p* value	OR [95% CI]; *p* value
No variant	1.73 [0.87–3.38]; *p* = 0.11	0.99 [0.96–1.02]; *p* = 0.64	0.98 [0.91–1.06]; *p* = 0.64
Bipartite iliac bony plate	0.14 [0.06–0.31]; ***p* < 0.001**	0.99 [0.96–1.02]; *p* = 0.419	0.95 [0.89–1.01]; *p* = 0.13
Accessory joint	0.51 [0.22–1.17]; *p* = 0.11	1.02 [0.98–1.05]; *p* = 0.41	1.01 [0.94–1.09]; *p* = 0.82
Iliosacral complex	2.53 [1.38–4.64]; ***p* = 0.003**	1.02 [1–1.05]; *p* = 0.103	1.07 [1.00–1.14]; ***p* = 0.04**
Semicircular defect	0.67 [0.23–1.94]; *p* = 0.46	0.95 [0.91–0.99]; ***p* = 0.01**	1.02 [0.93–1.11]; *p* = 0.74
Crescent iliac bony plate	0.79 [0.42–1.47]; *p* = 0.45	0.98 [0.95–1.01]; *p* = 0.22	0.93 [0.87–0.99]; ***p* = 0.03**
Isolated synostosis	3.4 [0.56–20.63]; *p* = 0.18	1.11 [1.03–1.2]; ***p* = 0.006**	0.92 [0.78–1.09]; *p* = 0.33
Unfused ossification center	3.34 [0.21–53.8]; *p* = 0.4	0.86 [0.76–0.98]; ***p* = 0.02**	0.95 [0.77–1.17]; *p* = 0.61
Dysmorphic posterior part	0.22 [0.81–0.61]; ***p* = 0.003**	1.01 [0.98–1.04]; *p* = 0.49	1.03 [0.94–1.13]; *p* = 0.51
Extra SIJ facet superiorly	*	0.88 [0.86–0.9]; ***p* < 0.001**	1.18 [1.11–1.25]; ***p* < 0.001**

Significant *p* values are marked in bold

SIJ = sacroiliac joint; BMI = body mass index; OR = odds ratio; 95% CI = 95% confidence interval

* = not estimable

Isolated synostosis was significantly more prevalent with advancing age, in contrast to semicircular defect and unfused ossification center, which were significantly more frequent in younger patients (Table 3).

The occurrence of iliosacral complex and the new variant with an extra SIJ facet superiorly was significantly more frequent in patients with higher BMI (Table 3). However, the variant with an extra SIJ facet superiorly was only present in one patient. In contrast, crescent iliac bony plate was significantly more frequent in patients with lower BMI.

### Combinations of multiple variants

**Fig. 4:**
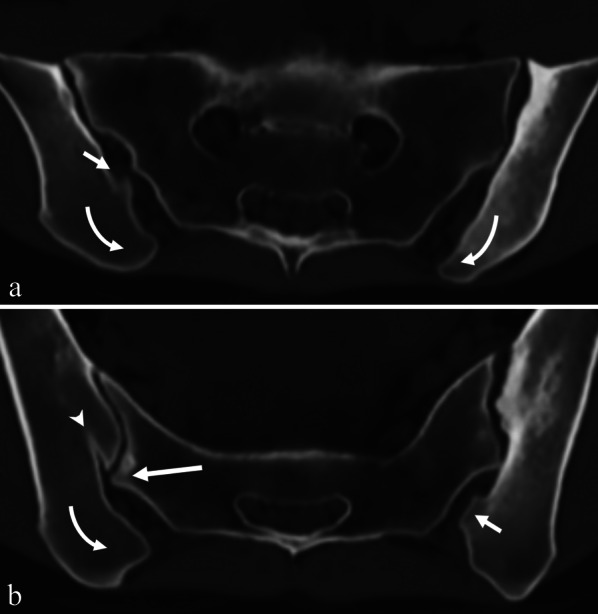
Multiple sacroiliac joint (SIJ) variants coexisting in one patient. Semi-axial (**a**) and axial (**b**) synthetic CT images of multiple coexisting variants in a 27-year-old woman. An accessory SIJ is visible posterior to the cartilaginous part of the SIJ on both sides (short arrow), as well as crescent iliac bony plate (curved arrow). Additionally, dysmorphic SIJ (long arrow) accompanied by degenerative subchondral cyst and bipartite iliac bony plate (arrowhead) are present on the right side. Also note erosions and marked sclerosis on the left side

**Fig. 5:**
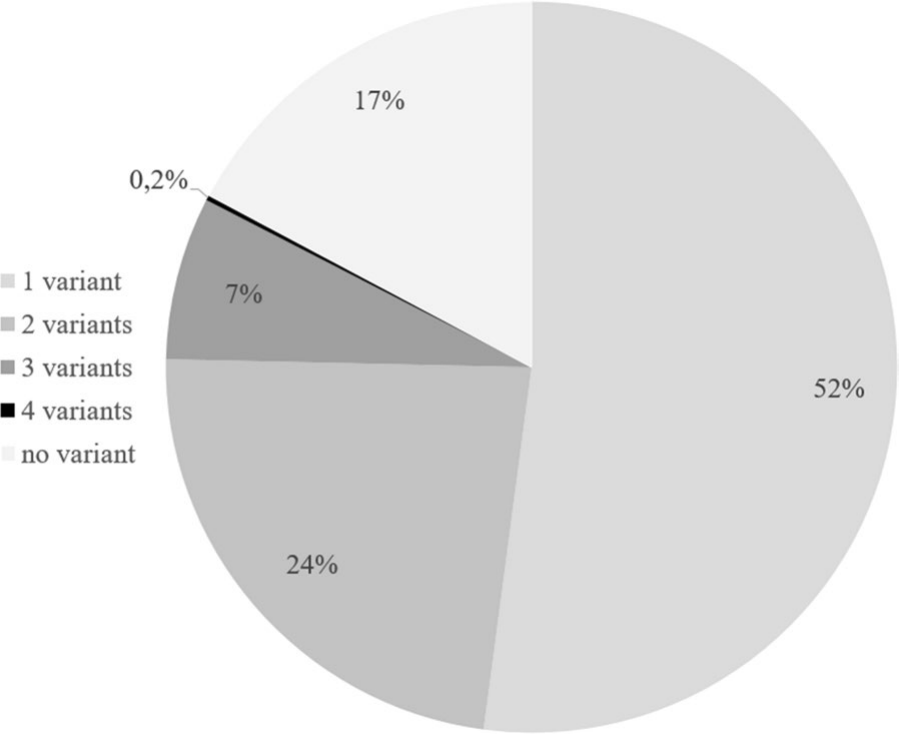
Pie chart of the number of variants that were found per sacroiliac joint

Most variants occur bilateral (Table 4). However, many combinations exist. We found coexistence of up to four variants within one joint (Fig. 4). Combinations of three variants were found in 31 SIJ (7.21%), and coexistence of two variants was found in 100 SIJ (23.26%) (Fig. 5). Taking into account the low frequency of some variants (isolated synostosis, unfused ossification center, and the newly found variant), almost all possible combinations were found in our population, except for iliosacral complex and semicircular defect.

**Table 4:** Bilateral existence and coexistence of multiple variants within one SIJ, analyzed for 430 SIJ

	Total incidence (*n* = 430)	Bilateral presence	Not in combination with another variant in one SIJ	Combination with at least one other variant within the same SIJ								
				Bipartite iliac bony plate	Accessory joint	Iliosacral complex	Semicircular defect	Crescent iliac bony plate	Isolated synostosis	Unfused ossification center	Dysmorphic posterior part	Extra SIJ facet superiorly
No variant	74	78.4% (58/74)										
Bipartite iliac bony plate	117	87.2% (102/117)	28.2% (33/117)	–	18.8% (22/117)	13.7% (16/117)	10.3% (12/117)	33.3% (39/117)	0.9% (1/117)	0.9% (1/117)	18.8% (22/117)	0
Accessory joint	56	75.0% (42/56)	30.4% (17/56)	39.3% (22/56)	–	8.9% (5/56)	1.8% (1/56)	26.8% (15/56)	5.4% (3/56)	0	14.3% (8/56)	0
Iliosacral complex	119	97.5% (116/119)	67.2% (80/119)	13.4% (16/119)	4.2% (5/119)	–	0	5% (6/119)	2.5% (3/119)	0	15.1% (18/119)	0
Semicircular defect	34	88.2% (30/34)	50% (17/34)	35.3% (12/34)	2.9% (1/34)	0	–	20.6% (7/34)	0	0	8.8% (3/34)	0
Crescent iliac bony plate	116	96.6% (112/116)	46.6% (54/116)	33.6% (39/116)	12.9% (15/116)	5.2% (6/116)	6% (7/116)	–	2.6% (3/116)	0	12.1% (14/116)	0
Isolated synostosis	9	66.7% (6/9)	0	11.1% (1/9)	33.3% (3/9)	33.3% (3/9)	0	33.3% (3/9)	–	0	0	0
Unfused ossification center	3	66.7% (2/3)	66.7% (2/3)	33.3% (1/3)	0	0	0	0	0	–	0	0
Dysmorphic posterior part	65	89.2% (58/65)	29.2% (19/65)	33.8% (22/65)	12.3% (8/65)	27.7% (18/65)	4.6% (3/65)	21.5% (14/65)	0	0	–	0
Extra SIJ facet superiorly	2	100.0% (2/2)	100% (2/2)	0	0	0	0	0	0	0	0	–

Values are N (number of observations), with row percentages in parentheses. Note that in some variants, the percentages do not add up to 100% because some SIJ demonstrated multiple variants, whereas others did not

SIJ = sacroiliac joint, *n* = number of observations

### Inter-reader variability for SIJ variants

The inter-reader agreement was moderate to good for unfused ossification center, semicircular defect and crescent iliac bony plate (K-values were 0.54, 0.73 and 0.79, respectively), very good (K-values ≥ 0.8) for bipartite ilium and accessory joint, and even excellent (with K-value ≥ 0.94) for iliosacral complex, dysmorphic joint and isolated synostosis. Agreement was perfect for detection of the new variant (extra SIJ facet superiorly), with a K-value of 1. The inter-reader reliability for not detecting any variant was also very good (K-value of 0.8).

## Discussion

In this study, we investigated the prevalence of variant forms of the SIJ using sCT images in patients with clinical suspicion of sacroiliitis. We found these variants to be very common in our study group: 82.8% of the investigated joints demonstrated at least one variant. This prevalence is much higher than in previous studies performed with CT and/or MRI, which reported overall incidences of 25.7–57% [1–3, 6, 7, 9]. The most frequent variants were iliosacral complex, bipartite iliac bony plate and crescent iliac bony plate (27.7, 27.2% and 27%, respectively). This is in line with the results of Tok [6] and Cihan [7]. Other authors found other variants to be the most frequent: accessory joint [2, 4, 5], dysmorphic cartilaginous joint [1, 3], or bipartite iliac bony plate [9].

These differences in prevalence can at least in part be explained by differences in study groups. In contrast to previous studies, we did not exclude patients with imaging findings of sacroiliitis [1–7]. Moreover, our patients were clinically suspected of sacroiliitis. This is in line with the findings of Ziegeler et al. [8], who also reported higher frequencies of atypical joint forms in symptomatic patients than in controls. They found SIJ variants in 80.3% of patients with mechanical SIJ disease, and 44.1% in patients with axSpA [8].

The high incidence of variant SIJ forms could further be explained by differences in imaging technique. The sCT images evaluated in this study consist of a data set with a slice thickness of 0.8 mm, of which reconstruction in any plane is possible. This allows for detection of very small or subtle findings, including variant forms of the SIJ. In contrast, previous CT- and MRI-based studies used different imaging protocols, often with thicker slices (up to 10 mm) and/or fixed slice orientation (e.g., only strictly axial images) [1–8]. Despite our imaging protocol with very thin slices, we found very low prevalences of isolated synostosis and unfused ossification center, similar to previous studies [1–9], indicating these are truly rare variants. Finally—in contrast to most other reports [3–7]—we allowed for more than 1 variant to be present in each SIJ, which also contributes to higher frequencies.

SIJ variants were often visible on the conventional MRI sequences as well. The T1-weighted sequence is best suited to discern these variants, as this is the most ‘anatomic’ sequence, allowing the best differentiation between cortical bone and surrounding tissues. T1-weighted spin echo without fat suppression is also the best suited conventional sequence to detect structural lesions of sacroiliitis including erosions and sclerosis [23]. However, in our experience, fewer SIJ variants were detected on the conventional MRI sequences. This is probably at least partially due to the slice thickness of 3 mm for the T1-weighted sequence, versus 0.8 mm for the sCT images. Another factor is the fixed semi-coronal plane of the available T1-weighted images, because in our experience, some variants can readily be seen in the semi-coronal plane of the sacrum (accessory SIJ, iliosacral complex, semicircular defect, isolated synostosis), while other variants are not or very difficult to depict in the (semi-)coronal plane as opposed to the (semi-)axial plane (bipartite iliac bony plate, crescent ilium, unfused ossification center, dysmorphic sacrum).

Some variants were even more frequently found than joints without SIJ variants. The very high prevalence raises the question if these variants—which are often regarded as abnormal—are actually subtypes of the ‘normal’ SIJ. We also found a new variant, never reported before, consisting of a supplementary extension of the SIJ on the posterior-superior side. It is possible that the classification used in the present study is still incomplete. The clinical significance of SIJ variants remains debated. Some authors demonstrated statistically significant associations between SIJ variants and degenerative changes [6, 9], and between SIJ variants and BME [1, 3, 10], whereas others could not find such associations [7]. Variations in SIJ form presumably can alter or aggravate biomechanical stress [1, 4, 6, 8, 9]. It is not clear if SIJ variations can cause symptoms in this way: further research is needed on this matter.

Almost every possible combination of variants was present in our population, but concomitant presence of iliosacral complex and semicircular defect was not found. It is possible that these two variants represent two ends of a spectrum: both essentially exhibit a focal groove in the sacrum, with an accompanying ilial prominence in iliosacral complex, but not in semicircular defect of the sacrum.

Overall, variants were slightly more often found in women than in men (85.8% and 77.8%, respectively), except for iliosacral complex, isolated synostosis and unfused ossification center. This trend is consistent with previous studies, although the difference is much smaller in our population [1–3, 6, 8, 9]. The finding that variants were common both in men and women does not support the hypothesis of pregnancy and child birth as an etiological factor in these variants [5].

Isolated synostosis was significantly more frequent with advancing age. On the other hand, semicircular defect and unfused ossification center were more frequent in younger patients—however, these variants were not very frequent in our study group; therefore, results could be due to overfitting. Other authors also found a higher prevalence of several variants in older patients [2, 5, 8]. These findings support the hypothesis that variants are not congenital but rather develop during life [2, 5]. The inversed relationship for unfused ossification center could indicate that these centers can still fuse to the SIJ later in life.

We investigated the relationship between SIJ variants and body weight and found that iliosacral complex was more frequent in patients with higher BMI, but a crescent joint form was more frequent in patients with lower BMI. Demir et al. found slightly different results compared to our study; they found a higher incidence of iliosacral complex in obese patients as well, but they also demonstrated this relationship for accessory SIJ and semicircular defect [5]. This could indicate a role for body weight in the existence and potentially development of the SIJ variants (possibly by altering biomechanical stress), further supporting the hypothesis of an acquired nature of different SIJ variants.

Our study has some limitations. We included patients in a tertiary university hospital, all clinically suspected for sacroiliitis, which can induce selection bias and limit generalizability of our results. Furthermore, we did not use strict definitions or provide measured requirements of the SIJ variants, e.g., depth of sacral defect or bipartite iliac bony plate. Also, it can be challenging to differentiate certain variants from pathologic alterations, for example to differentiate isolated synostosis (consisting of focal bony bridging) from acquired focal bony bridging due to sacroiliitis. This can lead to over- or underdiagnosis of SIJ variants. However, we believe we cannot set a threshold for these variants yet, as the clinical relevance (and thus the potential required size of any variant to induce certain effects) still remains debated. Another limitation is the varying inter-reader agreement. Although it was good to excellent for most SIJ variants, it was only moderate for the variant unfused ossification center. The use of more specified definitions could also lead to better inter-reader agreement. Finally, our study group consisted of a relatively limited amount of patients.

## Conclusion

SIJ variants are very common in patients suspected for sacroiliitis, and multiple variants can coexist within one SIJ. sCT enables detection of very small or subtle findings including SIJ variants. The clinical significance of these variants remains unclear.

## Data Availability

The datasets generated and analyzed during the current study are not publicly available because the subjects did not provide written consent for their data to be publicly shared.

## Abbreviations

axSpA: Axial spondyloarthritis
BMI: Body mass index
CI: Confidence interval
CT: Computed tomography
GEE: Generalized estimating equations
K: Kappa
MRI: Magnetic resonance imaging
OR: Odds ratio
PACS: Picture archiving and communication system
sCT: Synthetic computed tomography
SIJ: Sacroiliac joint
ST: Slice thickness
STIR: Short tau inversion recovery imaging
TE: Echo time
TR: Repetition time
